# Toll-like receptor 1/2 activation reduces immunoglobulin free light chain production by multiple myeloma cells in the context of bone marrow stromal cells and fibronectin

**DOI:** 10.1371/journal.pone.0310395

**Published:** 2025-01-28

**Authors:** Jahan Abdi, Frank Redegeld

**Affiliations:** 1 Department of Clinical Science, CHHSN, California State University Dominguez Hills, Carson, California, United States of America; 2 Division of Pharmacology, Department of Pharmaceutical Sciences, Faculty of Science, Utrecht University, Utrecht, The Netherlands; Dana Farber Cancer Institute, Harvard Medical School, UNITED STATES OF AMERICA

## Abstract

Toll-like receptor (TLRs) activation in multiple myeloma (MM) cells induces heterogeneous functional responses including cell growth and proliferation, survival or apoptosis. These effects have been suggested to be partly due to increase in secretion of cytokines such as IL-6 or IFNα among others from MM cells following TLR activation. However, whether triggering of these receptors also modulates production of immunoglobulin free light chains (FLCs), which largely contribute to MM pathology, has not been investigated in MM cells before. This study explored the effect of TLR1/2 ligand (Pam3CSK4) alone or combined with bortezomib (BTZ) on production of FLCs in human myeloma cell lines, L363, OPM-2, U266 and NCI-H929. It also investigated the above effect when MM cells were exposed to bone marrow stromal cells (BMSCs) or fibronectin (FN). Adhesion to BMSCs or FN increased secretion of FLC in MM cells. Pam3CSK4 decreased FLC production, and this effect was enhanced in combination with BTZ but attenuated when MM cells adhered to BMSCs or FN. The findings of this study imply that activation of TLR1/2 downregulates FLC production in MM cells even in the context of bone marrow microenvironment components and suggest that targeting some TLRs such as TLR1/2 might have therapeutic potential.

## Introduction

Within the bone marrow microenvironment, multiple myeloma (MM) cells closely interact with BMSCs or extra cellular matrix proteins such as FN, collagen, laminin, and tenascin [1, 2]. This interaction plays a key role in maintaining the survival and drug resistance of MM tumor cells, mediated mostly by direct contact (adhesion) or secretion of a plethora of cytokines and growth factors by BMSCs or MM cells, including IL-6, VGEF, IGF-1, HGF, IL-3 and RANK [3, 4]. These cytokines are also known to contribute to angiogenesis and osteolytic bone lesions in MM [3, 4].

MM cells are dependent on microenvironmental cues to survive [5, 6]. As potential microenvironmental cues, Toll-like receptor (TLR) ligands have been suggested to play important modulatory roles in growth, proliferation and viability of MM cells which display a high but heterogeneous expression pattern of these receptors [7–12]. Our previous study demonstrated that activation of TLR1/2 modulated expression of adhesion molecules on MM cells and their adhesion to BMSCs [13]. This observation prompted us to hypothesize that TLR activation in MM bone marrow microenvironment also modulates secretion of immunoglobulin free light chains (FLCs) which are known to contribute significantly to MM complications [14, 15].

The biologic pattern of FLC production has not been fully explored in MM experimental models following drug treatment. In light chain myeloma accounting for 20% of MM patients, when secretion of FLCs exceeds the maximum re-absorptive capacity of renal tubular system, in situ protein complexes will form resulting in nephropathy [16]. Therefore, targeting mechanisms controlling the production of FLCs by the malignant clone would be an efficient therapeutic approach.

This study demonstrates that FLC production is enhanced in human myeloma cell lines (HMCLs) following their adhesion to FN or BMSCs, and Pam3CSK4 (alone or combined with bortezomib) reduces FLC production by HMCLs in the presence or absence of these bone marrow microenvironment components. Bortezomib (BTZ) is a first-generation proteasome inhibitor which has been applied to clinical trials of MM and mantle cell lymphoma [17]. While the results indicate that TLR/1/2 activation inhibits FLC production in MM cells, further studies are required to uncover the molecular mechanisms underlying adhesion- and TLR-mediated modulation of FLCs in MM cells.

## Materials & methods

### Cell lines and cell culture

Human multiple myeloma cell lines (HMCLs), L363, OPM-2, U266, and NCI-H929, were provided by Hematology department of Utrecht University Medical Center [18, 19] and were maintained in Roswell Park Memorial Institute-1640 medium (RPMI-1640, no HEPES, ThermoFisher Scientific) supplemented with 5–10% FBS and 2mM glycine (referred to as complete medium), and intermittently with antibiotics. The human bone marrow stromal cell line, HS-5, was obtained from American Type Culture Collection (ATCC) and maintained in Dulbecco’s Modified Eagle Medium (DMEM, high glucose, ThermoFisher Scientific) supplemented with 10% FBS (complete medium), and intermittently with antibiotics.

### Chemicals

TLR1/2 ligand (Pam3CSK4) was obtained from Invivogen and dissolved in sterile water according to manufacturer’s instructions to make a 1 mg/ml stock. Bortezomib (BTZ) was from LC Laboratories and was dissolved in dimethyl sulfoxide (DMSO)as a 100mM stock. The final DMSO concentration never exceeded 0.01% in all experimental conditions. Human plasma-derived fibronectin (FN) was from Sigma.

### Cell stimulation

For cell stimulation we followed a sequential treatment procedure: First, HMCLs were harvested from cultures and incubated with a range of Pam3CSK4 doses (0.05–2.5μg/ml) for 24h. Then, cells were washed and re-suspended in fresh RPMI complete medium and seeded (or not) on FN- or BMSC-coated wells of a 96-well plate. In some experiments, a specific dose of BTZ was added to the wells containing HMCLs pre-activated with Pam3CSK4 and incubation continued for another 24h (see below).

### Co-culture of MM cells with HS-5 cells

To assess FLC production of HMCLs following adhesion to BMSCs and to see the effect of Pam3CSK4 on FLC secretion by HMCLs in this context, HS-5 cells (100,000 cells) were first seeded on 12-well plates and incubated to achieve 70% confluency in almost 40h as explained before [13]. Then, HMCLs pre-activated for 24 h with different concentrations of Pam3CSK4 (0.05–5.0μg/ml) were washed, re-suspended in serum-free RPMI and added to HS-5 containing wells (one million cells to each well) and incubated for 2h. Contents of the wells were gently aspirated by tilting the plate and wells were gently washed once with warm RPMI to remove unattached MM cells. Fresh complete RPMI medium was added to each well and plates were further incubated for 24 h. At the end of the incubation, supernatants were collected and assayed for FLC concentrations using ELISA as explained below. In separate experiments, to examine the combined effect of BTZ+Pam3CSK4 on FLC production in HS-5 context, HMCLs pre-stimulated with Pam3CSK4 were washed with warm RPMI and incubated with 1μM of BTZ in protein-containing medium for one h (*acute exposure*), washed again to remove the drug and resuspended in warm drug-free medium, seeded on HS-5 cells as explained above and incubation was extended to 24 h followed by collection of the supernatants for FLC analysis.

### Culture of HMCLs on FN-coated microwells

To investigate whether FLC production of HMCLs stimulated (or not) with Pam3CSK4 is modulated by adhesion to FN (an important extra-cellular matrix protein within BM microenvironment), 12-well plates were coated with 20μg/ml FN overnight at 4°C. Plates were blocked with sterile heat-denatured BSA (10mg/ml in PBS) for 1 h at room temperature and washed with sterile PBS. Following our previous protocols [13, 20] with slight modification, HMCLs (1×10^6 cells) pre-activated with Pam3CSK4 (or unstimulated cells) as described above were washed using warm RPMI, re-suspended in serum-free RPMI and added to each coated well for 1 h. Unattached cells were removed by gentle aspiration and one-time washing using warm RPMI, and the wells were further incubated with fresh complete RPMI medium for 24 h. In separate experiments, to assess the combined effect of BTZ+Pam3CSK4 on FLC production in FN context, BTZ (5nM) in complete RPMI medium was added after removing the unattached cells and incubation extended to 24 h (*chronic exposure*). FLC level in the supernatants of all experimental conditions was measured at the end.

### ELISA

Kappa or lambda FLCs were assayed as previously described [21]. Briefly, 96 well plates were coated overnight at 4°C with goat anti-mouse IgG antibody in bicarbonate buffer. Subsequently, plates were blocked using blocking buffer (1% BSA in PBS) for 1 h (RT) followed by incubation with mouse-anti human kappa or lambda Ig-FLC mAbs (obtained from Dr. A. Solomon, Tennessee) for 1h. Next, plates were incubated with different dilutions of samples and standards (The Binding Site), washed and incubated with HRP-labelled goat F(ab’)2 anti-human kappa or lambda Ig light chain Abs (AHI1804 and AHI1904, respectively, Biosource, USA). Finally, the reactions were developed using tetramethylbenzidine (TMB) and measured through an ELISA plate reader (BioRad).

### Statistical analyses

We found two-way ANOVA in GraphPad software to be the best statistical method for data analysis and generating the graphs based on this logic: there are two main groups (adhered and non-adhered) and within each group several independent factors (Pam3, BTZ, Pam3+BTZ) impact the outcome (FLC reduction, dependent factor), also all the data from both groups need to be compared with each other. *p*<0.05 was considered as significant.

## Results

### Baseline production of FLC by HMCLs

First, baseline concentration of FLC in all the cell lines was measured and it was found that the cell lines showed a heterogenous pattern of LC isotypes. As shown in Table 1, L363, OPM2, U266, were lambda (λ) light chain producers, and NCI-H929 was a kappa (κ) light chain producer.

**Table 1:** Production of different light chain isotypes by HMCLs at baseline.

Cell line	Light chain isotype
L363	Lambda
OPM-2	Lambda
U266	Lambda
NCI-H929	Kappa

### TLR1/2 activation in HMCLs reduces FLC secretion

**Fig 1:**
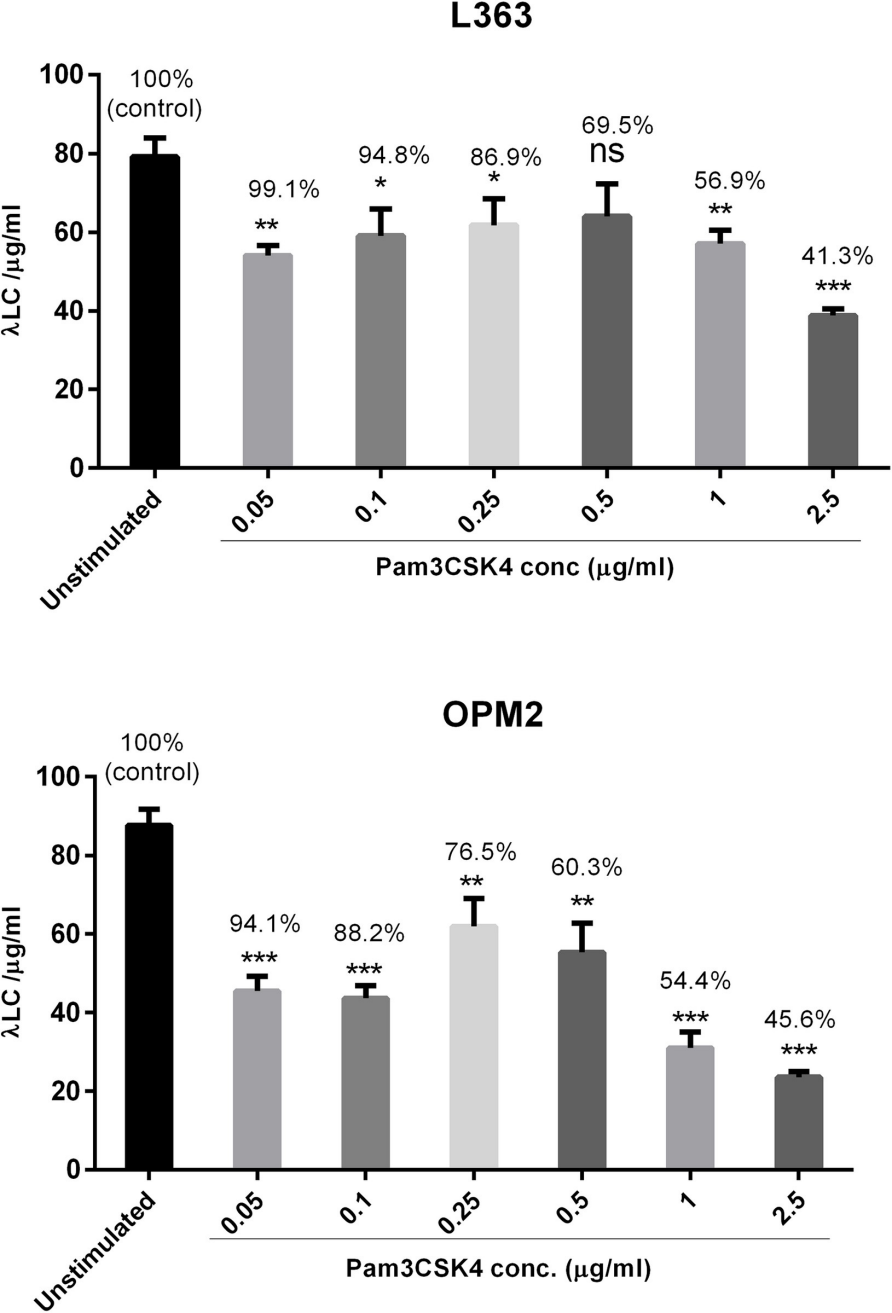
Effect of Pam3CSK4-induced cell death on FLC production by HMCLs. L363 and OPM2 cells were stimulated with a wide range of Pam3CSK4 concentrations (0.05–2.5μg/ml) for 24 h. Supernatants were collected for FLC analysis, and cells were applied to PI FACS staining to determine % viability of cells and whether FLC production was affected by cell death (% on top of the bars indicate viability of cells in each condition).

TLR activation in MM cells has been associated with heterogenous effects on these cells including modulation of cytokine secretion particularly IL-6 which mediated MM cell proliferation and survival following TLR triggering [8, 9]. However, whether TLR activation might also modulate FLC secretion of MM cells in the presence or absence of FN or BMSCs (HS-5 cells) was the main question asked in this study. To this aim, we treated L363, OPM-2, U266 (lambda light chain producers) and NCI-H929 (kappa light chain producer) cells with a range of Pam3CSK4 concentrations (0.05–5.0μg/ml) for 24h and FLC levels were measured in culture supernatants (**Fig 1**). However, since our previous study showed that Pam3CSK4 would induce apoptosis in HMCLs at 2.5μg/ml or higher [13], we investigated if the inhibitory effect of Pam3CSK4 on FLC production was due to increased cell death. To this aim, for L363 and OPM2 cells we determined % viability of cells related to each Pam3CSK4 concentration using propidium iodide staining followed by flow cytometry. Depicted in **Fig 1**, all Pam3CSK4 doses reduced FLC production in both cell lines. Although all doses of Pam3CSK4 displayed inhibitory effect on FLC secretion, the overall response pattern looked bell-shaped. Interestingly, the 3 doses (0.05, 0.10 and 0.25 μg/mL) resulted in significant reduction in FLC secretion while the viability of MM cells was 76–99% indicating that contribution of cell death to FLC reduction was unlikely or negligible. However, at doses above 0.25μg/mL while FLC was significantly reduced dose-dependently, the viability of MM cells also strikingly declined. Based on this observation, it would be safe to assume that at lower end of the range (0.05–0.25μg/ml) Pam3CSK4 specifically reduces FLC production, but at higher doses FLC reduction is mostly due to cytotoxicity. We used the 0.05μg/mL dose of Pam3CSK4 in all other experiments.

### Adhesion to BMSCs or FN increases secretion of FLCs in HMCLs

**Fig 2:**
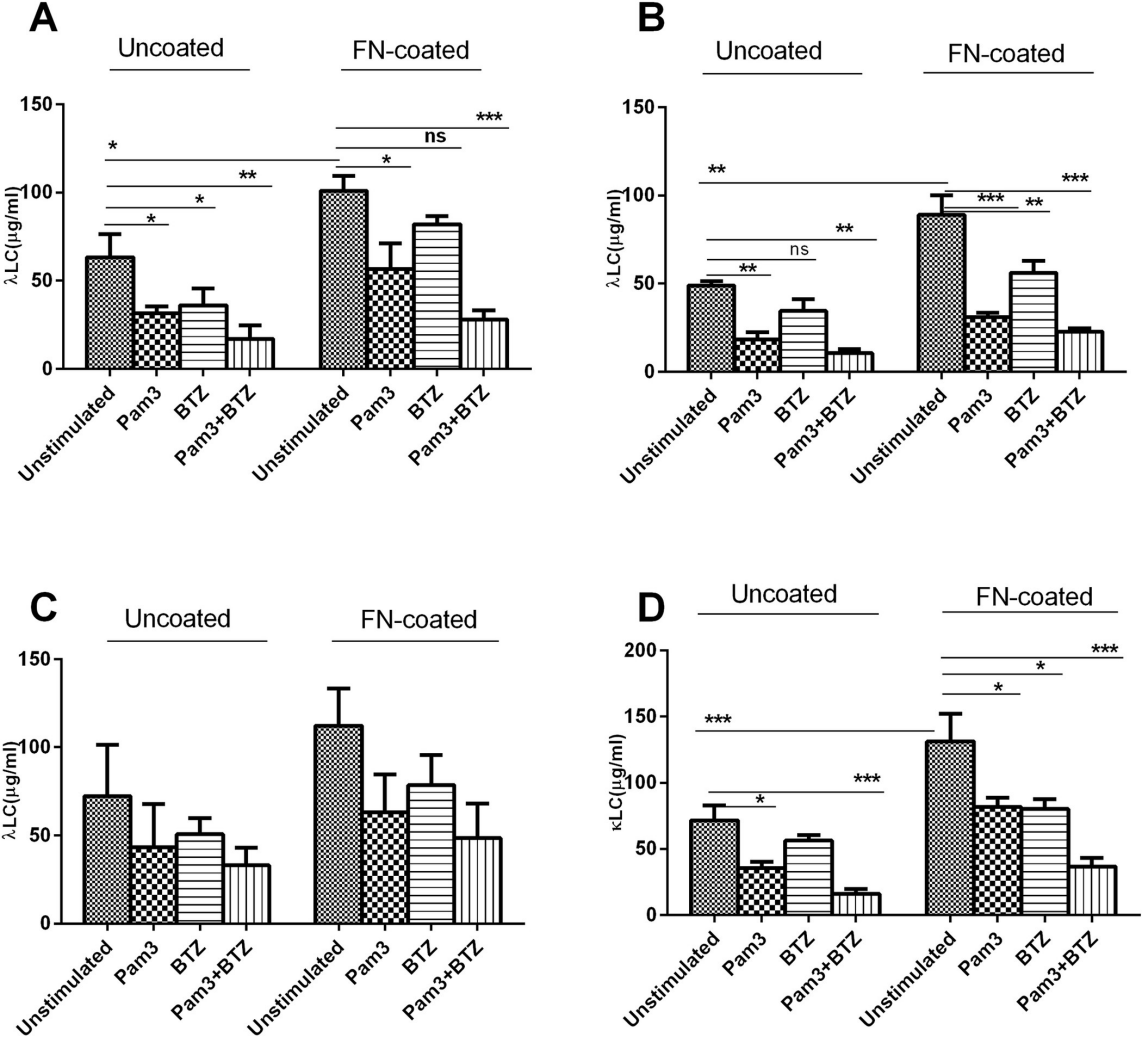
The effect of Pam3CSK4 on FLC production in HMCLs: λ in L363 (**A**), OPM-2 (**B**), U266 (**C**), and κ in NCI-H929 (**D**) after exposure to BTZ in the context of FN. HMCLs were stimulated (or not) with 0.05μg/ml Pam3CSK4 for 24 hours, washed, adhered (or not) to FN and exposed (or not) to 5nM BTZ for 24h as explained in materials and methods. Data are the mean±SD from analysis of three separate experiments. * *p < 0*.*05*, ** *p <0*.*01*, *** *p <0*.*001*.

**Fig 3:**
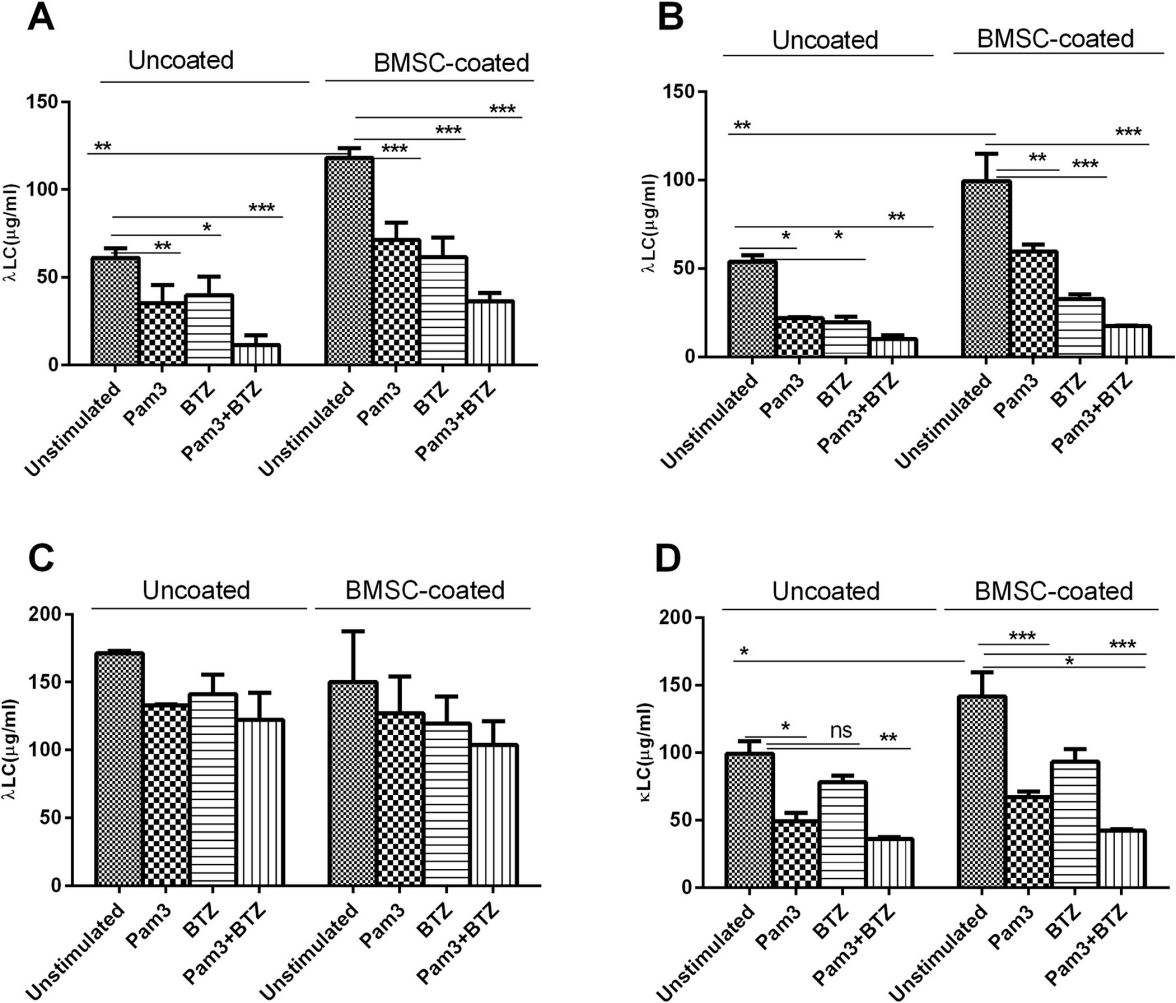
The effect of Pam3CSK4 on FLC production in HMCLs: λ in L363 (**A**), OPM-2 (**B**), U266 (**C**), and κ in NCI-H929 (**D**) after exposure to BTZ in the context of BMSCs. HMCLs were stimulated (or not) with 0.05μg/ml Pam3CSK4 for 24 h, washed, adhered (or not) to BMSCs and exposed (or not) to 1μM BTZ for one h (more explanation in the text). Data are the mean±SD from analysis of three separate experiments. * *p < 0*.*05*, ** *p <0*.*01*, *** *p <0*.*001*.

Adhesion of MM cells to extracellular matrix proteins such as FN or to BMSCs is reported to induce proliferation and survival of MM cells and render them resistant to drugs, an observation originally termed cell adhesion-mediated drug resistance (CAMDR) [22, 23]. Whether such adhesion also modulates secretion of FLC by MM cells is not clear. To address this question, we co-cultured HMCLs with HS-5 cells or cultured on FN-coated microwells for 24 h. As shown in **Fig 2**, L363 (A), OPM-2 (B), U266 (C) and NCI-H929 (D) cells *unstimulated* with Pam3CSK4 significantly increased their production of FLC following adhesion to FN (uncoated vs coated). Likewise, in **Fig 3** the same cell lines *unstimulated* with Pam3CSK4 significantly increased their production of FLC after adhesion to HS-5 cells (uncoated vs coated). However, U266 cell line displayed inconsistent pattern in HS-5 context compared to other cell lines, and in FN context while adhesion-induced increase in FLC production was obvious, the difference between uncoated and coated conditions did not reach significant level. These observations imply that bone marrow microenvironment components including BMSCs and FN might play a regulatory role in FLC secretion by MM cells through adhesion-mediated signaling, however, further mechanistic studies are required to support this.

### Pam3CSK4-induced reduction in FLCs is enhanced in combination with BTZ in the presence or absence of bone marrow microenvironment

Adhesion of MM cells to bone marrow stroma components such as FN and BMSCs induces secretion of various cytokines in MM cells and renders them resistant to drugs [3, 23]. Having shown that the above adhesion upregulates FLC production in HMCLs, we sought to explore whether Pam3CSK4 alone or in combination with BTZ would affect FLC production by HMCLs while these cells are adhered to FN or BMSCs. Again, as shown in **Figs 2 and 3**, BTZ significantly decreased FLC production in L363, OPM2 and NCI-H929 cell lines, in the presence or absence of FN or BMSCs, but this inhibitory effect of BTZ was not significant in U266 cells. Also, comparison between uncoated and coated conditions in all cell lines shows that the inhibitory effect of BTZ or Pam3CSK4 is slightly attenuated in adhered cells due to the impact of BMSCs or FN. Combined treatment with Pam3CSK4+BTZ showed at least an additive effect in reducing FLC production although in adhered cells this inhibitory effect was also lower. Furthermore, to see if FLC reduction by BTZ is not due to cell death, in a separate experiment 10000 (arbitrary number) cells each of L363 and OPM2 cell lines were exposed to a dose range of BTZ (0.25–5.0nM) for 24h in a 96-well plate. At the end of incubation, the number of live cells for each dose was determined in parallel with FLC measurement (S1 Fig). The results imply that FLC reduction by BTZ doses of 0.25–1.0nM could not be due to cell death but at 2.0nM and higher the effects of cell death on FLC production cannot be ignored. Taken together, our findings indicate that Pam3CSK4 (TLR1/2 activation) in HMCLs diminishes FLC production in the presence or absence of bone marrow microenvironment components and this effect would be enhanced in combination with BTZ at least in an additive fashion.

## Discussion

This study is the first to report that TLR activation in MM cells modulates FLC secretion in these cells. It showed that Pam3CSK4 significantly decreased FLC production by all HMCLs. Pam3CSK4 is a synthetic triacylated lipopeptide (LP) and a TLR1/2 ligand. It is a potent activator of the pro-inflammatory transcription factor NF-κB [24, 25]. Pam3CSK4 mimics the acylated amino terminus of bacterial LPs. At the cell surface, TLR2 forms a heterodimer with co-receptors TLR1 or TLR6, depending upon either tri- or diacylation of the ligand. Once a ligand binds to either TLR2-TLR1 or TLR2-TLR6, a MyD88-dependent activation of NF-κB and AP-1 occurs, ultimately leading to an innate immune response. However, recognition of Pam3CSK4, a triacylated LP, is mediated by TLR2 which cooperates with TLR1 through their cytoplasmic domain to induce the signaling cascade leading to the activation of NF-κB [26].

Studies have shown that the expression pattern of TLRs and functional outcomes of their activation in MM cells is quite heterogenous. For instance, activation of TLR4 and TLR9 in MM cells promoted expression of anti-apoptotic genes BCL-2 and MYC [27], or enhanced secretion of IL-32 which is reported to be associated with poor prognosis in MM [10]. On the contrary, TLR3 activation induced proliferation of some HMCLs (KMM1) but suppressed growth of others (NCI-H929, RPMI 8226) [28]. We demonstrated previously that TLR1/2 activation (Pam3CSK4 treatment) in MM cells displayed heterogenous effects on the expression of adhesion molecules (αVβ3, α4, β7 integrins) and adhesion of these cells to BMSCs and FN [13, 20]. It remains to be determined whether activation of different signaling pathways could explain different TLR activation outcomes in MM cells.

Our previous study also showed that Pam3CSK4 at 2.5μg/ml and higher induced MM cell death and sensitized MM cells to BTZ [13, 20]. Here Pam3CSK4 at lower doses (0.05–0.25μg/ml) significantly reduced FLC production by MM cells with minimal effects on cell viability, but toxic effects gradually appeared at doses above 0.50μg/ml although FLC production was also decreased significantly. Of note, Pam3CSK4 was shown to have oncogenic effect in gastric carcinoma cells and TLR2 targeting inhibited gastric tumorigenesis [29]. On the contrary, consistent with the findings of this study, Pam3CSK4 synergized with Ara-C in killing B cell lymphoma cells and it triggered apoptosis of acute myeloid leukemia (AML) cells indicating possible context-dependent activation effects of TLR1/2 activation [30, 31].

Cell adhesion molecules (especially integrins) play fundamental role in BMSC- or FN-mediated proliferation and survival of MM cells and their protection against anti-myeloma drugs hence cell adhesion mediated drug resistance (CAMDR) [3, 32–37]. In the present study, MM cells upregulated their FLC production following adhesion to FN or BMSCs implying involvement of adhesion-mediated signaling pathways in FLC modulation. Importantly, integrin-mediated adhesion of plasma cells to intestinal epithelial cells is critical for maintaining IgA-dependent mucosal immune protection [38]. Furthermore, Pam3CSK4 effectively reduced FLC level in the presence or absence of bone marrow microenvironment components, FN and BMSCs, and this effect was significantly enhanced in combination with BTZ at least additively. However, the extent of FLC reduction in adhered cells (FN and BMSCs) was lower compared to non-adhered cells implying that FLC inhibitory effects of BTZ or BTZ+PamCSK4 could be hampered due to increased secretion of FLC by MM cells in the presence of bone marrow stroma.

## Conclusions

This study provides new insights into the impact of TLR1/2 activation on FLC production by MM cells and their pattern of FLC production in the presence of bone marrow microenvironment components. It also shows that the inhibitory effects of Pam3CSK4 alone or in combination with BTZ on FLC production in MM cells could have therapeutic potential. It remains to be explored mechanistically how FLC secretion is regulated in MM cells following their adhesion to BMSC or FN (role of integrin-mediated signaling) and whether activation of other TLRs (TLR4, 5, 7,8,9) also impacts FLC secretion by MM cells. Understanding these mechanisms will help identify new therapeutic targets in MM microenvironment. Finally, our study suggests further investigation into pre-clinical application of Pam3CSK4 alone or in combination with BTZ in MM *in vivo* studies.

## Supporting information

S1 Fig Effect of BTZ-induced cell death on FLC production in MM cells. 10000 cells each of L363 and OPM2 cell lines were exposed to a dose range of BTZ (0.25–5.0nM) for 24h in a 96-well plate. At the end of incubation, the number of live cells for each dose was determined in parallel with FLC measurement.
(TIF)

S1 File (XLS)
