# Supplementary figures and images for: Toll-like receptor 1/2 activation reduces immunoglobulin free light chain production by multiple myeloma cells in the context of bone marrow stromal cells and fibronectin
Source: PLoS One. 2025 Jan 28;20(1):e0310395. doi: 10.1371/journal.pone.0310395 (PMC11774389; doi:10.1371/journal.pone.0310395)

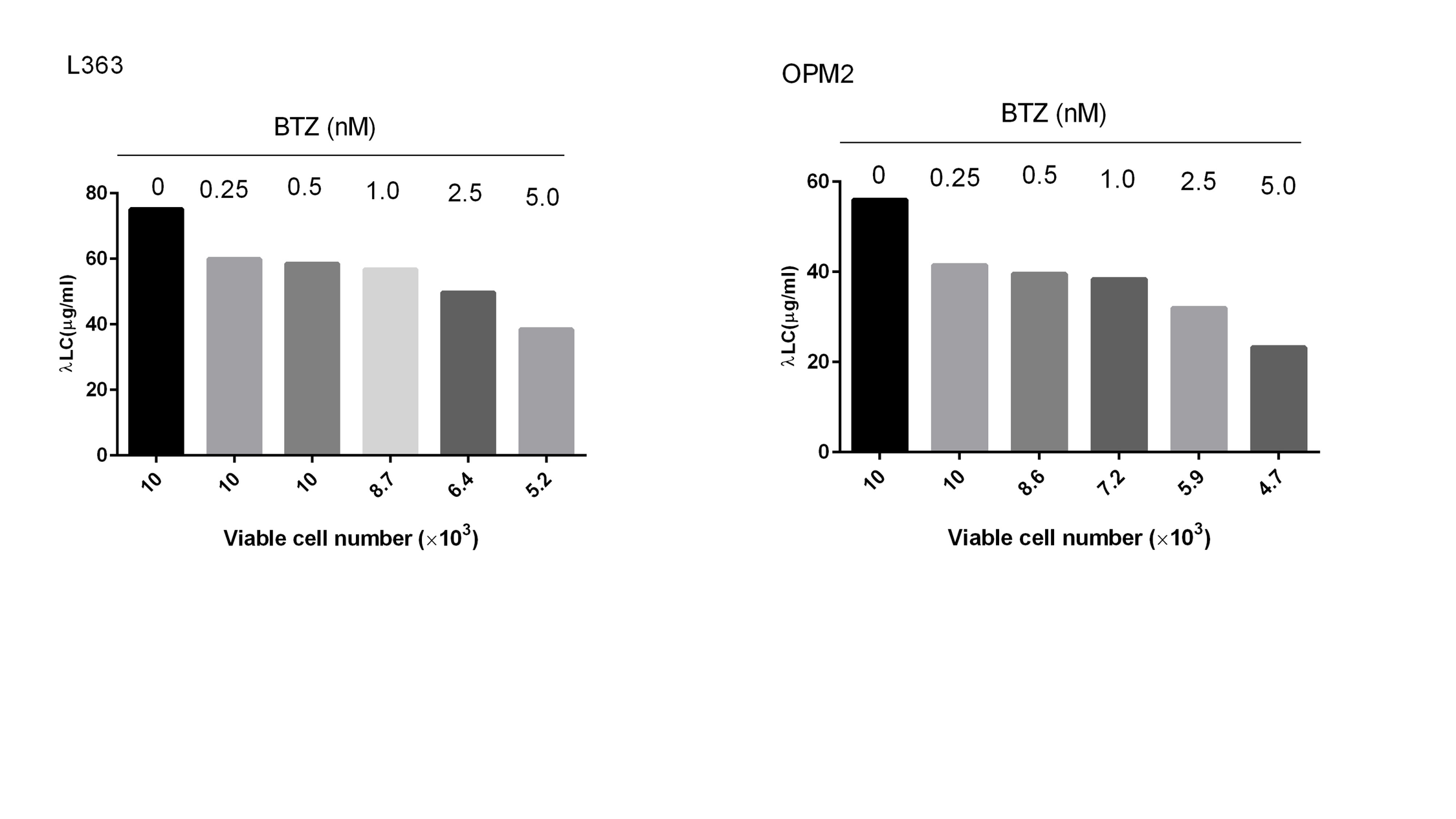

Supplement: S1 Fig — 10000 cells each of L363 and OPM2 cell lines were exposed to a dose range of BTZ (0.25–5.0nM) for 24h in a 96-well plate. At the end of incubation, the number of live cells for each dose was determined in parallel with FLC measurement. (TIF) [file pone.0310395.s001.tif]
